# Multiple independent analyses reveal only transcription factors as an enriched functional class associated with microRNAs

**DOI:** 10.1186/1752-0509-6-90

**Published:** 2012-07-23

**Authors:** Larry Croft, Damian Szklarczyk, Lars Juhl Jensen, Jan Gorodkin

**Affiliations:** 1 Center for Non-coding RNA in Technology and Health, Division of Genetics and Bioinformatics, IBHV, University of Copenhagen, Copenhagen, Denmark; 2 NNF Center for Protein Research, Faculty of Health Sciences, University of Copenhagen, Copenhagen N, Denmark

## Abstract

**Background:**

Transcription factors (TFs) have long been known to be principally activators of transcription in eukaryotes and prokaryotes. The growing awareness of the ubiquity of microRNAs (miRNAs) as suppressive regulators in eukaryotes, suggests the possibility of a mutual, preferential, self-regulatory connectivity between miRNAs and TFs. Here we investigate the connectivity from TFs and miRNAs to other genes and each other using text mining, TF promoter binding site and 6 different miRNA binding site prediction methods.

**Results:**

In the first approach text mining of PubMed abstracts reveal statistically significant associations between miRNAs and both TFs and signal transduction gene classes. Secondly, prediction of miRNA targets in human and mouse 3’UTRs show enrichment only for TFs but not consistently across prediction methods for signal transduction or other gene classes. Furthermore, a random sample of 986 TarBase entries was scored for experimental evidence by manual inspection of the original papers, and enrichment for TFs was observed to increase with score. Low-scoring TarBase entries, where experimental evidence is anticorrelated miRNA:mRNA expression with predicted miRNA targets, appear not to select for real miRNA targets to any degree. Our manually validated text-mining results also suggests that miRNAs may be activated by more TFs than other classes of genes, as 7% of miRNA:TF co-occurrences in the literature were TFs activating miRNAs. This was confirmed when thirdly, we found enrichment for predicted, conserved TF binding sites in miRNA and TF genes compared to other gene classes.

**Conclusions:**

We see enrichment of connections between miRNAs and TFs using several independent methods, suggestive of a network of mutual activating and suppressive regulation. We have also built regulatory networks (containing 2- and 3-loop motifs) for mouse and human using predicted miRNA and TF binding sites and we have developed a web server to search and display these loops, available for the community at http://rth.dk/resources/tfmirloop.

## Background

Transcription factors (TFs) have long been known to be the predominant regulators of transcription in eukaryotes and prokaryotes, on the whole serving as activators of transcription. In the last 10 years a growing awareness of the ubiquity of miRNAs as suppressive regulators in eukaryotes has emerged [1]. Though miRNA regulation is ubiquitous, knockdown of the miRNA pathway produces limited or unobservable phenotypes in some cell lineages [2-4]. This limited effect, coupled with the small change in abundance of the average mRNA targeted by a miRNA (for example in Selbach *et al.*[5]), lead to the suggestion miRNAs act in the majority of cell lineages to fine tune mRNA expression in a dynamic, regulatory manner, and thereby increase the robustness of the regulatory network [6].

Previous work has shown that miRNAs preferentially target TFs more than other gene classes [7-14], and the miRNAs themselves also appear to be regulated by TFs more than other types of gene [15]. Thus, it appears there is an interconnected network of mutual regulation between miRNAs and TFs. Analysis of these networks for recurring motifs identified feed forward [16-21] and feedback motifs [18,22], both as 2-element and 3-element loops. A 2-element loop being 2 nodes mutually connected by directed edges, and 3 element loops being 3 nodes where all nodes are connected to the others by edges of either direction.

These loops appear to be important functional elements. For example 2 coupled feedback loops (LMO2, miR-223, miR-363) are necessary for normal hematopoiesis (Major personal communication), and it has been suggested these loop motifs may confer robustness to genetic regulatory networks [6,23-26], presumably by inhibiting stochastic transcriptional perturbation using feedback and feed forward loops to regulate transcriptional “gain” and control amplification of unwanted frequencies much as these loops are used in mechanical and electrical engineering for example [27-29]. The ability of these loops to confer robustness was elegantly demonstrated experimentally in Drosophila development [19]. Li *et al.* previously demonstrated removal of *mir-7* had no observed phenotypic effect in Drosophila sensory cells under standard laboratory conditions [30], just as many miRNA knockouts or knockdowns have no observable phenotype. However when Li *et al.* perturbed embryos by regularly varying the temperature, *miR-7* mutant flies had abnormal sensory cells, demonstrating that the hybrid feed forward/feedback motif containing *miR-7* imparted robustness to sensory cell development. Li *et al.* surmised miRNAs may be often used to impart robustness to regulatory networks, and this may explain the apparent lack of phenotype of many miRNA knockouts in standard laboratory settings. Hilgers *et al.*[31] found similar miRNA regulatory motifs, where *mir-263a* and *mir-263b* mutants displayed a reduced number of mechanosensory eye bristles, which they suggest is due to damage to an incoherent feedforward loop controlling apoptosis of progenitor cells.

How common are these loop motifs in regulatory networks? Is the limited phenotype of many miRNA knockdown/outs due to the prevalence of robustness-conferring miRNA:TF loops or other more complex regulatory structures? Are miRNAs less important than other classes of gene, or is their role less critical? Otherwise, is there built-in redundancy in the miRNA regulatory network of which we are unaware? Given the reports of enrichment for feedforward and feedback motifs in genetic regulatory networks [32-35], and the observation of these motifs in critical cellular pathways [36-39], we also touch on the presence and role of combined TF:miRNA regulatory networks including 2 and 3 element loop motifs. To our knowledge a comprehensive search for all statistically significantly enriched 3-element loop motifs has not been done before. In this analysis we do not include chromatin regulation, which is likely to be a pervasive system of suppressive regulation in the metazoan genomes. Only a small number of loci are well documented, and these utilise diverse, DNA-protein, RNA-DNA and RNA-protein specific interactions, beyond the scope of this work.

Motivated by the potential for miRNAs and TFs to act in interlinked regulatory networks we address the above questions, establishing miRNA and TFs connections from independent sources. This paper is organized as follows; first we employ text mining to find any potential functional gene class associated with miRNAs in the literature. For the next approach we employ 6 miRNA target prediction methods and following that we employ a transcription factor binding site prediction method. We extract information derived from these combined resources, and describe a resource of predicted miRNA:TF interactions in 2- and 3- element loop motifs which we make available to the community.

## Results

### Text mining demonstrates enrichment for miRNA:TF links

**Figure 1:**
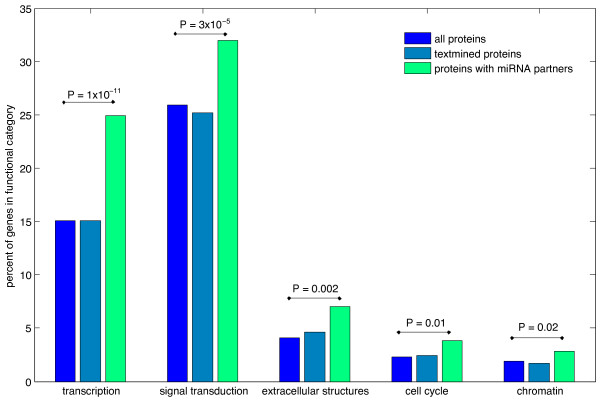
**Abundance of text-mined proteins co-occurring with miRNAs in the literature by functional category.** All text-mined proteins compared to proteins co-occurring together with miRNAs in the literature, grouped by eggNOG functional category. P-values are calculated from a Fisher Exact test comparing all proteins to proteins which co-occur with miRNAs.

Using the text-mining pipeline of STRING [40] with dictionaries of protein (STRING 8.3) and miRNA (miRBase 15) names, we identified all 1800 pairs of human protein-coding genes co-occurring with miRNAs (see Methods for details). We searched for statistical enrichment of functional categories [41] from eggNOG 2.0 [42] and also GO terms [43] for proteins co-occurring with miRNAs. To avoid text-mining bias when estimating enrichment the frequencies of all text-mined proteins in STRING were taken as a background or control set (Figure 1), with enrichment for miRNA targeted proteins shown as a percentage compared to the control set.

Two functional categories demonstrate significant enrichment: transcription and signal transduction (see methods and additional tables: Text mining by eggNOG categories and text mining by GO). The extracellular structures category was also enriched, though this was found to be due to overlap with the signal transduction category. The transcription category is heavily represented by TFs, while the signal transduction category predominantly contains upstream components of transcriptional activation, such as receptors and signalling kinases. The signal transduction category also contains a small number of TFs induced by such signalling kinases. The potential of signal transduction pathways to produce feedforward and feedback loops with miRNAs and TFs will not be covered here, but is discussed in [44]. Our results in the next section suggest signalling pathway elements, apart from TFs involved in signalling, are not consistently enriched for miRNA binding sites compared to other genes. To further verify our text-mining results we randomly selected and manually annotated 300 of the miRNA:protein pairs using the same literature evidence collected for the text mining. We counted the number of miRNAs suppressing TFs, the number of TFs activating miRNAs, and the number of literature co-occurrences which did not contain regulatory linkages. This dataset reiterated our previous text-mining results and also highlighted the large number of TFs known to regulate miRNAs published in the literature.

Our results demonstrated a surprisingly high linkage in the literature between miRNAs and TFs, as also observed in [8]. It is possible the literature is biased towards links between miRNAs and TFs due to the common interest in these two classes of genes. To test this we compared computationally predicted miRNA target site occurrences in TF and non-TF genes.

### miRNA target site prediction

**Figure 2:**
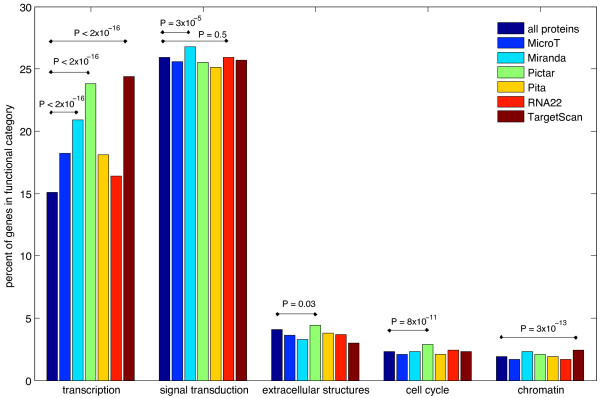
**Abundance of 6 different miRNA target prediction methods by functional category.** All proteins compared to 6 miRNA target prediction methods’ proteins by eggNOG functional category. P-values are calculated from a Fisher Exact test comparing all proteins to each methods’ predicted target proteins.

**Figure 3:**
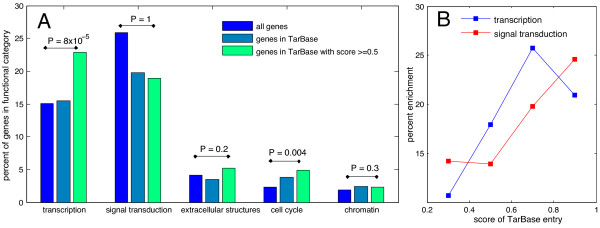
**Functional enrichments for high scoring TarBase genes.** (**A**) All TarBase genes compared to scored TarBase genes by eggNOG functional category. (**B**) Enrichment for transcription and signal transduction functional categories by score of TarBase source literature. P-values are calculated from a Fisher Exact test comparing all genes to high scoring TarBase genes.

Predicted miRNA target sites from RNA22 [45], TargetScan [46], MiRanda [47], MicroT [48], PITA [49] and PicTar [50] were collected (see methods). Most of these methods utilise RNA binding energy and binding site conservation for prediction, so they are of use as literature independent tests of enrichment. TFs were enriched for predicted miRNA binding sites in all prediction methods (Figure 2) compared to non TF genes. There was no consistent enrichment for signal transduction or any other gene classes. This suggests that the enrichment seen for signal transduction in the text-mining dataset was due to bias for publication of miRNAs targeting signal transduction genes, a result we see also in the TarBase (version 5) dataset of experimentally validated miRNA binding sites (Additional file 1: Table S1 TarBase by eggNOG categories and text mining by eggNOG categories), but only when we use a high quality subset of TarBase. The complete TarBase dataset shows no enrichment for TFs (Figure 3A). However after manually scoring 986 TarBase entries from 1 to 0, based upon the strength of published experimental evidence for each miRNA:mRNA interaction, where 0 would be no experimental evidence and 1 is a completely certain miRNA target site, an enrichment for TFs is seen with a score cutoff of 0.5. Increasing the score cutoff increases the enrichment (Figure 3B), but also shrinks the sample size. miRNA binding site scores below 0.5 are chiefly computationally predicted miRNA binding sites coupled with anticorrelated miRNA:mRNA expression. It seems this is unlikely to be sufficient evidence to enrich for real miRNAs to a significant degree. The large expression datasets and the high false positive rate of miRNA target prediction create many instances of anti-correlated expression of a miRNA:mRNA and predicted target site by chance.

Enrichment of TFs for miRNA targets has been observed in other studies [7-9] as well as our own. Enrichment for signalling kinases (and kinase inhibitors) has also been observed [8]. As the TF enrichment is present in predicted miRNA targets, literature bias is not the principle cause, and the enrichment demonstrates a real tendency for miRNAs to target TFs.

### Comparison of miRNA target site prediction methods

If enrichment for predicted miRNA binding sites in TF genes is due to a biological predisposition for miRNA regulation of TFs, this can be used to compare miRNA target prediction methods. Figure 2 demonstrates MiRanda, PicTar and TargetScan have increasing enrichment for miRNA binding sites in TFs (but not consistently in other classes), suggesting increasing true positive rates for these prediction methods.

### TFBS prediction

**Figure 4:**
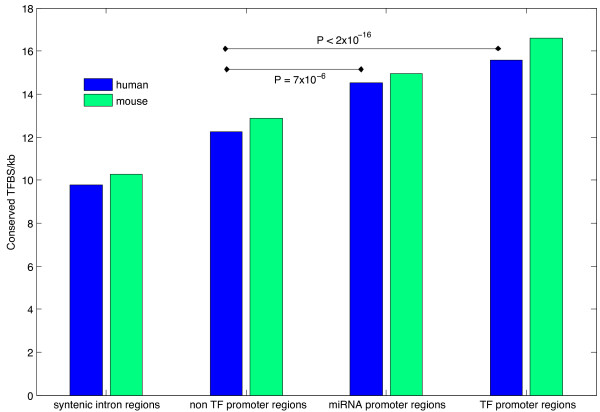
**Conserved transcription factor binding site densities in mouse and human promoter regions.** Conserved transcription factor binding site density for mouse and human transcription factor, non transcription factor and miRNA promoter regions compared to syntenic intronic regions. P-values are calculated from a Fisher Exact test comparing non TF promoter regions with miRNA and TF promoter regions.

Having shown miRNAs target TFs more frequently than other gene classes, and noting TFs are targeted by multiple miRNAs more than other gene classes [9], we investigated whether miRNA promoters contain more TFBS than the average protein coding gene promoter. This was also suggested by the high frequency of TFs regulating miRNAs identified in our manual annotation of 300 miRNA:protein pairs (7%, see additional File 1: Table S1 Text-mining data). TFBS were predicted from the weight matrices in JASPAR [51](October 2009 version) using Fimo [52] to scan sequences within 1 kb of annotated miRNA promoter regions. JASPAR was chosen as the only TFBS dataset where supporting data for our results could be placed in the public domain. By counting the number of human-mouse conserved TFBS from the prediction we demonstrate that miRNAs and TFs have enrichments for TFBS compared to the 1 kb promoter regions of non TF genes, or 1 kb syntenic mouse-human intronic regions (Figure 4). Intronic regions were chosen as a control set for comparison as a number of miRNA and miRNA clusters are contained in introns. We chose the host gene promoter if the miRNA was inside a protein coding gene, and we clustered miRNAs which occurred within 500nt of each other and chose the 1 kb upstream of the first miRNA. Promoters from miRNAs in intergenic regions were chosen 1 kb upstream of the annotated miRNA, regardless of the unknown structure of the primary miRNA transcript. As a first and conservative approach we ignore possible promoters inside introns, since the presence of an intronic promoter does not rule out the intronic miRNAs also being transcribed from their host gene promoter.

As these chosen miRNA promoter regions on average had higher densities of TFBS compared to either promoter regions of protein coding genes or mouse-human syntenic intron regions, we infer that miRNA promoter regions are enriched for active TFBS, though there are potential confounding effects such as differences in GC content between genomic regions. We found differences in TFBS density due to different base compositions to be far smaller than the differences we see between miRNA, non TF genes and TF genes, though higher order sequence structure may also be a confounding effect, or a product of higher real TFBS density.

### miRNA:TF networks

By collecting the activating links of TFBS, and the suppressive links of miRNA binding sites, it was possible to build a directed, 2 coloured (suppressive and activating links) graph of the predicted human and mouse genetic regulatory networks (detail in methods). We searched for all 2 and 3-element loops and measured their enrichment compared to 3 different random network models. It is well known that genes can be targeted multiple times by the same miRNA, though the tissue specificity of each link is unknown, so when counting links it is unclear how to weight or reduce redundancy of multiple (predicted) target sites in the same mRNA. Therefore, with current experimental data, it is only possible to apply the following two strategies: (i) Include all multiple target sites (completely redundant), and (ii) a collapsed version (fully non redundant) where we only counted a single link between a gene and the miRNA, in spite of multiple (predicted) target sites. In the second case we identified no statistically significant enriched loop motifs. In the first case, we obtained enrichment for loop motifs. When considering each (predicted) target site as an individual link, we obtained a statistically significant enrichment for loops, however, this was due to redundant links being counted multiple times in a combinatorial manner in each loop. It remains unclear if a redundancy reduction reflecting the actual biological state would yield statistical significance. The search for enriched 3-loops was carried out by using Fanmod [53] which identified loops in networks of redundant and non redundant links (see Additional files 2 and 3).

**Figure 5:**
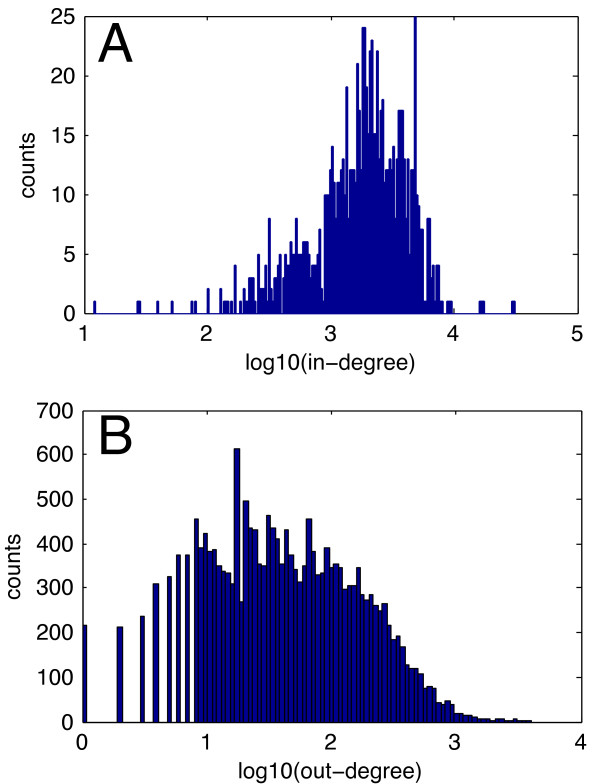
**In-degree and out-degree histograms for human TF:miRNA predicted networks.** (**A**) log10(in-degree) histogram for all human nodes in the TF:miRNA predicted network. (**B**) log10(out-degree) histogram for all human nodes in the TF:miRNA predicted network.

We observed the majority of loops identified in the human and mouse networks contain a non TF, non miRNA gene. Figure 5 shows histograms of the number of links into a node (in-degree) (Figure 5A) and the number of links out of a node (out-degree) (Figure 5B) for human. Most of the genome is linked within these networks. The peak of the in-degree histogram shows a typical regulator has the potential to influence thousands of genes, while each gene contains tens to hundreds of predicted TF and miRNA binding site. Obviously all genes contain TFBS, and many transcripts contain miRNA binding sites [54], however as each cell type expresses cell specific miRNAs and transcripts, the degree to which a gene is regulated by these links in a specific cell is unknown.

By counting the number of gene targets for the eggNOG transcription category for each miRNA, then dividing by the total number of gene targets for each miRNA, we could measure each miRNA’s tendency to target TFs. While the TF category was generally targeted by all miRNAs more often than other categories, individual miRNAs did not have strong tendencies to target TFs compared to the average across all miRNAs (data not shown).

To search for loops and genes within loops relevant for particular research applications we built a simple webserver, http://rth.dk/resources/tfmirloop for this purpose, where the individual 2- and 3- element loops are available, along with other network data, downloadable as raw tab delimited text files. Using the web database it is possible to identify loops containing genes of interest. For instance searching for loops containing the oncogene *HRAS* (*ENSG00000174775*), identifies a coherent feedforward loop potentially suppressing *HRAS* where *miR-892a* directly suppresses *HRAS* and, indirectly, suppresses HRAS by suppressing the oncogene *PLAG1*, a putative activator of *HRAS*. miRNA deregulation of *PLAG1* is already known to occur in leukemia cells [55], thus there is a possibility HRAS may in some circumstances be a downstream effector of *miR-892a* and *PLAG1*. This obviously needs further experimental investigation to verify the predicted regulatory links.

## Discussion

Given the regulatory roles of both miRNAs and transcription factors (TFs), it is of relevance to analyze how these might be components within the same networks. The first step is to establish which relationships exist between miRNAs and TFs. Here, we conducted three complete independent analyses of how genes in general might be associated with miRNAs and TFs. Interestingly, when combining the results of these a clear enrichment for miRNA and TF association stand out as the only association for which there is consensus. Our text mining verified miRNAs target TFs more than other gene classes. Using TF binding site (TFBS) and miRNA target site predictions we observe preferential, mutual interconnections between TFs and miRNAs, in accord with previous studies.

We also addressed if it was possible to build human and mouse genetic regulatory networks based upon predicted, conserved TFBS, and utilising three different methods of miRNA target site prediction. Our interest in regulatory loops and the advantage that statistically significant loop enrichment can be observed even against a background of high false positive rate link prediction, led us to search for 2 and 3-element loops.

In many cases there are multiple links between nodes, either many identical miRNA binding sites in a UTR, or several identical TFBS in a promoter. The possibility of these binding sites acting independently of each other in different cell types due to cell specific RNA structures and protein binding events, makes these links potentially biologically independent. As each link may be independently active in a given cell type, a non-redundant link network underestimates the number of loops possible in the regulatory network, while a redundant link network includes all possible loops, irrespective of whether a loop is physically possible in any cell. A significant number of redundant links will be due to false positives, so a redundant network may overestimate the number of loops, however comparing redundant random networks to predicted networks ameliorates this to some degree.

We applied two approaches giving upper and lower bounds to the number of possible loops. In the first redundant case we identified many enriched feedforward and feedback loops in the mouse and human networks. In the second case we searched for 3-element loops in non-redundant networks and observed no enriched motifs. The actual abundance of 3-element loops unfortunately lies somewhere between these two extremes. Until such time as there are surveys of cell type and tissue specific miRNA and TF binding data, it will remain an open question whether the mouse and human genetic regulatory networks contain enriched motifs such as 3-element loops or other more exotic structures. The enriched connections between miRNAs and TFs, and the experimental observation of regulatory loops of miRNAs and TFs suggest a complex structure, containing feedforward and feedback elements that we currently can only perceive indirectly and with difficulty.

## Conclusions

In conclusion we have identified preferential interconnectivity between TFs and miRNAs using several methodologies. Text mining finds enrichments for TFs and signalling kinases. There is an enrichment for predicted miRNA binding sites in TF 3’ UTRs and also enrichment for predicted TFBS in miRNA and TF promoter regions. Binding site predictions corroborate text mining, and demonstrate the mutual preferential connection between miRNAs and TFs. The suppressive and predominantly activating nature of these links may form a regulatory network robust against perturbation, as experimental evidence suggests robustness may be explained by miRNA:TF loops, containing feedback and feedforward using suppressive and activating links. Given the current, partial state of our knowledge, the observable enriched connections between miRNAs and TFs demonstrates how common these connections must really be. Shortly with PAR-CliP, ChIP-seq and other methods our knowledge of miRNA binding sites, TF binding sites, and chromatin state in many cell types will grow dramatically. This will make it possible to study in depth the regulatory architecture cells use to control the subtleties of body plan development, of which we are currently only partially aware.

## Methods

### Text mining

All 4050 miRNA and 7,723,077 protein references in all 19.6 million PubMed abstracts (as of September 2010) were extracted by the text-mining pipeline of STRING. We used a dictionary of miRNA and protein synonyms (see files at http://rth.dk/resources/tfmirloop/) to cover possible name variants. SQL was used to intersect abstracts containing both miRNA and protein identifiers. These miRNA: protein pairs were compared to computationally predicted miRNA binding site data, and a random subset of these pairs were manually annotated to estimate the number of TF= > miRNA, miRNA= > TF and spurious links. The fraction of links in each category from the random subset gave an estimate of the different classes in the entire set with sufficient accuracy for the purposes of this study.

### Verification of text mining

As the text-mining pipeline only identifies literature co-occurrence, we randomly chose 300 miRNA:protein pairs and their original supporting PubMed abstracts, were randomly chosen from the 1800 pairs for manual validation. Each abstract was read and pairs were categorised as either miRNA suppresses protein, protein (TF) activates miRNA, miRNA: protein co-occur but were not functionally related or protein or miRNA is misidentified in the text mining. In 22% of the pairs either the miRNA or the protein was an artifact of text mining. For example the plasmid mir-1 is easily confused with the miRNA mir-1 and in other cases proteins have names which are common words in the English language. 36% (108/300) of the pairs were miRNAs having good experimental evidence for targeting the mRNA encoding the protein. 7% (22/300) of pairs were TFs that regulate expression of miRNAs.

### miRNA target site prediction

PITA, PicTar, MicroT, TargetScan, MiRanda and RNA22 miRNA target predictions for human and mouse were downloaded from their respective websites and reformatted to Ensembl identifiers (Release 57) using BioMart and Perl scripts. To detect the functional enrichment of targeted genes we employed two classification sets 1) functional categories of primate orthologue groups (prNOGs from eggNOG v2.0) with an exclusion of “general” and “unknown” categories (23 in total) 2) All Gene Ontology (version GO201010) terms under “molecular function” domain were collapsed to the highest discernable level excluding terms that did not render hits to any human proteins (13 in total). The Fisher exact test was used to calculate the enrichment of all above categories compared to their distributions in all protein-coding genes. For construction of the regulatory networks MiRanda, PicTar and TargetScan data were used as these three methods showed the highest enrichment for TFs, and hence we suspect the best true positive rate. Selection criteria and filtering cutoff parameters for each miRNA target prediction methodology can be found in the original documentation for each prediction method.

### Transcription factor binding site prediction

We used FIMO [52] to search for known motifs of TFBS 1kbp upstream of the Ensembl annotated [56] transcription start using default parameters (p-value < 10^-5^) and weight matrices (vertebrate core non-redundant set converted to MEME Minimal Motif Format [57] from JASPAR [51]. If the orthologous human and mouse regions both contained the same predicted TFBS, irrespective of the exact syntenic position, we added these TFBS to our predicted regulatory network. The position variability and difficulty of aligning such small, degenerate motifs precluded a more refined methodology. Care was taken to compare these 1kbp promoter regions against similar promoter regions to minimise different TFBS prediction rates due to base composition biases. Thus TF promoter regions were compared to non-TF protein-coding gene promoters, and miRNA promoter regions were compared to syntenic intron sequences. TFBS densities were compared between TF promoters, non TF promoters, miRNA promoters and syntenic intronic sequences using a Wilcoxon rank sum test to calculate p-values as the underlying distribution of TFBS is not normally distributed (Additional file 1: Table S1 TFBS density distributions). All obtained p-values were smaller than 1x10^-16^, suggesting a significant difference between the sets, given current biologically plausible assumptions. Random genomic regions were also used as a control set, and behaved identically to intronic sequences (data not shown).

miRNA promoter regions were collected by taking into account miRNA clusters and miRNAs within genes. miRNAs were clustered if they were within 500 bp of each other. Promoter regions were selected as 1kbp 5’ of the first miRNA in the cluster. Promoter regions for miRNAs within protein coding genes were taken to be the host gene’s promoter. While there is good evidence for miRNAs in introns being transcribed from their own promoter, or even alternately from the host gene promoter or its own promoter [58-60], we assumed conservatively (with respect to our analysis) the miRNA is driven from the host gene promoter.

### Web server

Motif and network data were loaded into MySQL (version 5.0.77) tables and a PHP server (version 5.2) was used to interface web side searches with the tables. The web server can be viewed at http://rth.dk/resources/tfmirloop.

## Competing interests

The authors declare that they have no competing interests.

## Authors’ contributions

DS and LC collected the data. LC, DS, LJ and JG designed the experiments. DS and LC analysed the data. LC wrote the paper and made the web server. All the authors read and approved the final manuscript.

## Supplementary Material

Additional file 1 **supplementary_tables.xlsx.** This file contains source data, additional graphs, and calculated values for this work in 15 Excel sheets: Text-mining data, text mining by eggNOG categories, text mining by GO categories, TarBase scoring system, TarBase data scored, TarBase score *vs.*TF enrichment, TarBase by eggNOG categories, TarBase by GO categories, predicted miRNA targets by eggNOG categories, predicted miRNA targets by GO categories, transcription factor binding site density distributions, human network 3-element motifs, mouse network 3-element motifs, human enriched loops, mouse enriched loops. Click here for file

Additional file 2 **human_miRNA_TF_net_ensg.tdf.** Tab delimited text file containing all links in the human predicted miRNA:TF network. First column is start node, second column is end node, last column is 1 or 2 depending whether the link is activating (1 transcription factor binding site) or suppressing (2 miRNA binding site). Gene identifiers are ENSEMBL IDs and miRBase IDs.(GZ 5440 kb) Click here for file

Additional file 3 **mouse_miRNA_TF_net_enmusg.tdf.** Tab delimited text file containing all links in the mouse predicted miRNA:TF network. First column is start node, second column is end node, last column is 1 or 2 depending whether the link is activating (1 transcription factor binding site) or suppressing (2 miRNA binding site). Gene identifiers are ENSEMBL IDs and miRBase IDs. (GZ 4011 kb) Click here for file
